# Vision–Language Model for Visual Question Answering in Medical Imagery

**DOI:** 10.3390/bioengineering10030380

**Published:** 2023-03-20

**Authors:** Yakoub Bazi, Mohamad Mahmoud Al Rahhal, Laila Bashmal, Mansour Zuair

**Affiliations:** 1 Computer Engineering Department, College of Computer and Information Sciences, King Saud University, Riyadh 11543, Saudi Arabia; 2 Applied Computer Science Department, College of Applied Computer Science, King Saud University, Riyadh 11543, Saudi Arabia

**Keywords:** medical visual question answering, vision–language encoders, transformer

## Abstract

In the clinical and healthcare domains, medical images play a critical role. A mature medical visual question answering system (VQA) can improve diagnosis by answering clinical questions presented with a medical image. Despite its enormous potential in the healthcare industry and services, this technology is still in its infancy and is far from practical use. This paper introduces an approach based on a transformer encoder–decoder architecture. Specifically, we extract image features using the vision transformer (ViT) model, and we embed the question using a textual encoder transformer. Then, we concatenate the resulting visual and textual representations and feed them into a multi-modal decoder for generating the answer in an autoregressive way. In the experiments, we validate the proposed model on two VQA datasets for radiology images termed VQA-RAD and PathVQA. The model shows promising results compared to existing solutions. It yields closed and open accuracies of 84.99% and 72.97%, respectively, for VQA-RAD, and 83.86% and 62.37%, respectively, for PathVQA. Other metrics such as the BLUE score showing the alignment between the predicted and true answer sentences are also reported.

## 1. Introduction

Assessing the physical state of the human body requires a variety of non-invasive sensory data. In particular, the use of medical imaging in disease diagnosis, screening, and surgical operations has prompted significant advances in this area [1,2]. Further, multi-modal data enable physicians to minimize diagnosis errors significantly [3,4]. One of the recent research topics in contemporary computer-aided diagnosis (CAD) is medical visual question answering (VQA) [5,6,7]. It involves both computer vision (CV) and natural language processing (NLP).

Typical medical image analysis systems include detection [8] and segmentation [9], which regard spatial information about objects and/or specific regions of interest across the image at hand. However, the need for ad hoc image analysis gave rise to sophisticated paradigms such as image captioning [10,11], which consists of building an informative textual caption about the image. For instance, in contrast to the limited mere object detection that produces spatial coordinates of objects in the image, image captioning takes it a step further by describing the relative position of objects, colors/textures, and the ongoing dynamics therein (e.g., a dog jumps over a hurdle, a kid is wearing a red hat, a guy is playing basketball, etc.).

In this regard, recent trends in computer vision attempt to drive image captioning into an even finer-grained version, namely visual question answering (VQA), by querying about detailed specifics about the image [12] (e.g., how many buildings are there at the top-right of the image? Are there cars parked on the roadside? etc.). Therefore, unlike the aforementioned image analysis approaches that require only an image as input, in VQA both the image and a relevant question are envisioned. Despite the fact that VQA has an interesting research basis in computer vision, thanks to the abundance of object annotations, in medical imaging it is still relatively lagging behind, and there is much room for improvement. 

A VQA pipeline normally consists of four blocks, namely (i) a feature extractor to derive visual features from the query image, (ii) a textual feature extractor from the query question, (iii) an embedding module that incorporates the former two modalities, and (iv) a prediction head. 

In this respect, the majority of existing medical VQA solutions rely on deep learning techniques such as recurrent neural networks (RNNs) [13] for text embedding and feature extraction, and convolution neural networks (CNNs) for visual feature extraction, as well as advanced techniques such as attention mechanisms. The advent of deep learning transformers has been effectively applied to the medical VQA task. For instance, transformers were first utilized in NLP tasks such as machine translation [14] and speech recognition [15]. Its encoder–decoder architecture is solely dependent on a self-attention mechanism. Unlike an RNN, which processes sequence items recursively and only pays attention to short-term context, transformers have demonstrated a potential to learn the relationships between sequence elements. Transformer designs may learn long-range associations by attending to whole sequences. In particular, the bidirectional encoder representation from transformers (BERT) [16] has been the most widely used model for representing textual information. BERT is a language model that employs a bidirectional attention mechanism with large-scale unsupervised corpora to provide a context-sensitive representation of each word in a phrase. 

The success of transformer networks in the context of NLP has attracted a lot of interest in computer vision. They were first proposed in [17], and have been successfully used in many tasks [18,19,20,21,22]. Further, they were adopted as a conventional transformer to images directly, by dividing them into patches that were treated as tokens in NLP applications [23,24,25]. 

However, despite the achievements related to vision-only and language-only tasks, there has not been much prior effort to connect multi-modal tasks with transformers [26,27,28,29,30,31]. Among these efforts, in [26], the authors introduced a UNiversal Image-TExt Representation (UNITER) that used a large-scale pre-trained model for combined multi-modal embedding. Hu et al. [27] proposed a unified transformer (UniT) based on encoding each input modality with an encoder and a joint decoder that makes predictions for multiple tasks including VQA. In contrast, VQA in the medical domain has not been validated yet by using multi-modal transformers.

Therefore, in this study we exploit transformer models for VQA in medical images. We devise a full transformer encoder–decoder architecture that takes medical images and relevant text as inputs and jointly uses them for training. This model consists of two encoders to encode each input modality. The extracted features from both modalities are fused using concatenation, followed by a decoder to draw the final answer predictions. With respect to previous literature on multi-modal learning with transformers, to the best of our knowledge, our work is the first that operates on medical images. Compared to the existing solutions, our model includes a decoder part for generating the open-ended answers in an autoregressive way.

The remainder of this paper is organized as follows: Section 2 reviews related works on VQA and the multi-modal transformer. Section 3 introduces the proposed VQA methodology. Section 4 presents detailed experimental analysis and presents comparisons with state-of-the-art methods. Finally, Section 5 draws conclusions and discusses future developments.

## 2. Related Work

In this section, we first provide an overview of the related works for VQA in the general computer vision and medical domains. Then, we discuss the multi-modal transformer architectures proposed in the literature.

### 2.1. Visual Question Answering 

With the success of deep learning, VQA in the general domain has received increasing attention in recent years. It has made tangible progress, mainly by exploiting deep CNN and RNN pipelines. For instance, many works rely on different RNNs such as LSTM [32], word2vec [33], and GloVe [34] for text embedding to capture the relationships between words and extract textual features. CNN architectures such as VGG16 [35], ResNet [36], and Faster R-CNN [37] were adopted for extracting low- and high-level visual cues. Afterwards, both feature representations were opportunely combined/fused to answer the query question by using more advanced attention techniques such as stacked attention networks (SANs) [38] and hierarchical co-attention [39].

VQA in medical domain tasks is derived from natural VQA tasks. Although some methods for natural images do not work effectively on medical images because of the different characteristics, some ideas and strategies from natural images are still beneficial to the medical field. However, in medical VQA, the development is much slower due to the lack of large-scale labeled training data, which require professional medical staff and a lot of time to carefully select and label high-quality relevant data. At this point, there are a few medical VQA datasets [40,41] that have been proposed. The VQA-RAD dataset [41] is one of the few datasets that provide data in the medical field of VQA. Many studies [6,42,43,44,45] developed their methods based on this dataset with manual annotations. For instance, the authors of [6] proposed a novel framework for learning reasoning skills conditioned on both question information and task type information. The authors of [43] utilized unlabeled radiology images to train teacher models via contrastive learning. The knowledge of the teacher models is distilled into a lightweight student model that is fine-tuned for medical VQA. Do et al. [43] presented a multiple meta-model quantifying method that learns meta-annotation and deals with noisy labels to provide robust features for medical VQA tasks. Pan et al. [44] proposed a multi-view attention-based model that gives attention to the question from two views, word-to-text and image-to-question, which helps to fuse the high-level semantics of images on the basis of text. The work in [45] adopted ResNet-34 [36] as a backbone which was jointly pre-trained on image understanding task and a question–image compatibility task. The model uses a cross-modal self-attention module for visual and linguistic feature fusion. PathVQA [40] is another dataset that contains more questions than other datasets in medical VQA. The work in [40] proposed bilinear attention networks (BANs) based on gated recurrent units (GRUs) [46] and a Faster R-CNN network for feature extraction. In [47], a model pre-trained with cross-modal self-supervised learning was devised to extract visual features. The model uses a learning-by-ignoring method to remove problematic training samples. In [48], an encoder–decoder architecture with a three-level optimization framework that relies on cross-modal self-supervised learning methods was developed to improve performance. Sharma et al. [49] proposed a model based on ResNet and BERT models with attention modules to focus on the relevant part of the medical images and questions. The model predicts the answer either by a classification or a generation head depending on the type of question. Finally, in [50], a bibranched model is proposed in which the first branch answers closed-ended questions with a transformer architecture, and the second branch answers open-ended questions with image retrieval that gives the most similar answer to the test image.

Despite the potential of these studies, medical VQA research is still lagging behind in terms of methodology (i.e., with regard to VQA in computer vision). Furthermore, the methodologies that have been proposed to date for medical VQA show limited performance and there is still much room for improvement.

### 2.2. Multi-Modal Transformer

Transformers were first applied in NLP tasks [51,52,53,54] and subsequently achieved satisfactory results in computer vision tasks. Most of the previous efforts on multi-modal learning focused on specific domains or single modalities. For instance, in [55] the authors applied an encoder–decoder architecture based on the transformer’s multi-head attention mechanism for image classification, machine translation, and image captioning. The work in [56] opts for a long-short transformer (Transformer-LS) with an efficient self-attention mechanism for both language and vision tasks. Tan et al. [30] utilized a method denoted as learning cross-modality encoder representations from transformers (LXMERT) that uses three transformer encoders with co-attention and only pre-trained the model with in-domain data. Lu et al. [29] used vision-and-language BERT (ViLBERT) with the same architecture but with more complex co-attention, and pre-trained with out-of-domain data. Despite the above-mentioned efforts, there are no existing works that attempt to tailor multi-modal transformers for medical VQA.

## 3. Methodology

**Figure 1:**
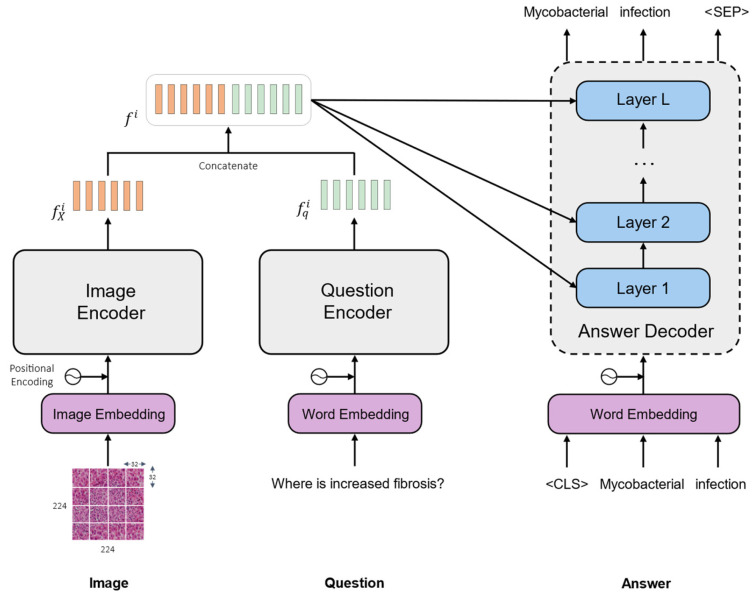
General architecture of the proposed medical VQA model.

Let us consider a set $D={\left\{X_{i},q_{i},y_{i}\right\}}_{i=1}^{N}$ composed of N triplets of a medical image, a question, and the corresponding answer. The goal is to teach the model to generate the correct answer $y_{i}$ to the question $q_{i}$ about the given medical image $X_{i}$. Figure 1 shows the overall framework of our medical VQA model, which is composed of an image encoder, a question encoder, and an answer decoder. 

Our model consists of a separate encoder for each input modality followed by a decoder. The first encoder is a transformer-based model that is used to extract visual features from the input medical image. The second is a language encoder that is used to generate a language representation from the input question. After encoding the two input modalities, the two feature representations are concatenated to generate a multi-modal representation from both the image and the question. Finally, the concatenated features go through several layers of the decoder to generate the proper answer. In the following, we describe the architecture of our framework in more detail:

### 3.1. Image Encoder

**Figure 2:**
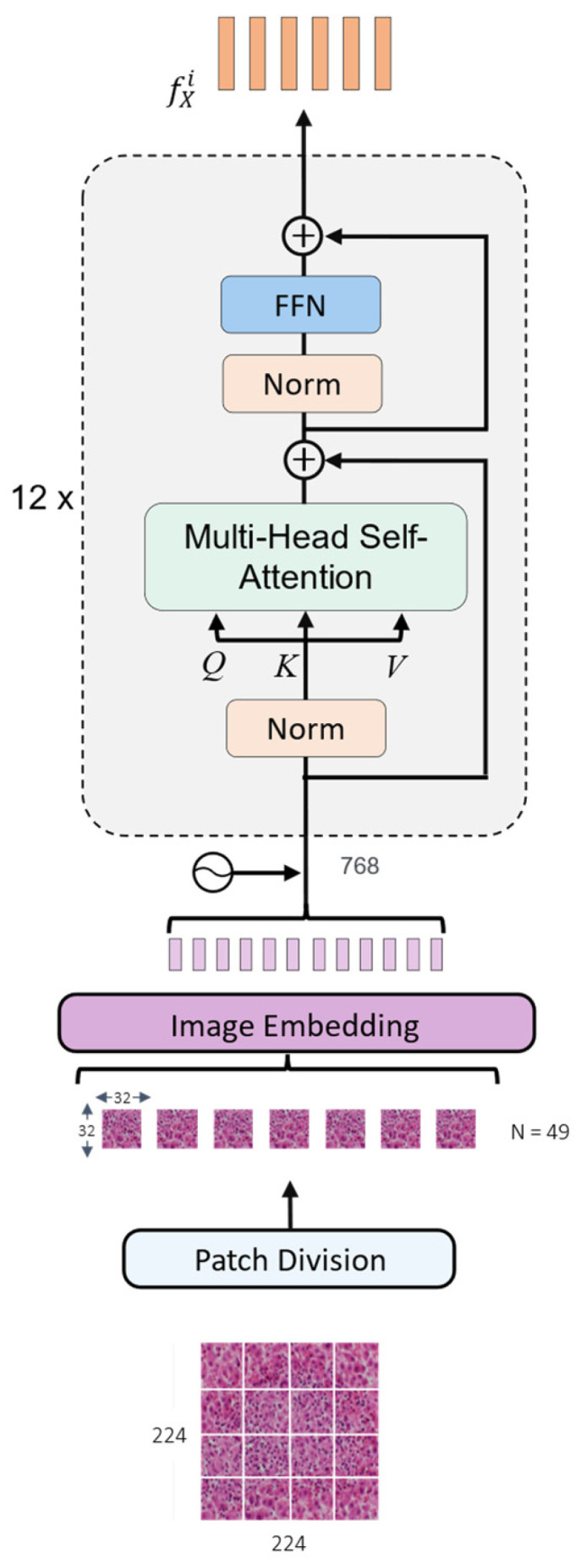
The architecture of the image encoder.

Figure 2 illustrates the detailed architecture of the image encoder. The medical image $X_{i}$ is fed into the image encoder to generate the corresponding visual representation. The image is first resized to the size of 224×224×3 pixels. Then, it is partitioned into 49 non-overlapping patches with a spatial dimension of 32×32 pixels. These patches are flattened into one-dimensional vectors and mapped with an image embedding layer into dimension 768 to match the encoder dimension. The positional encoding is combined with the patch representations and passed to the image encoder.

The adopted image encoder is a ViT32 model, which is a variant of the vision transformer proposed in [17]. Typically, this model is composed of 12 identical layers. Each layer comprises multi-headed self-attention (MSA) and feed-forward network (FFN) blocks that work together to generate visual features. Each block is preceded by a normalization layer [57] and a residual connection to the next block.

The MSA in the encoder employs the self-attention mechanism which is utilized to find correlations between different patches of the medical image. To determine this correlation, the embedded representation of the input image is transformed into three distinct matrices by using three linear layers. These resultant matrices are the query *Q*, the key *K*, and the value *V*. The dot product is calculated between the *Q* and the *K* matrices. The resulting value is divided by the square root of the dimension of the *K*. The score is passed through a SoftMax operation to obtain the attention weights. Finally, the *V* vector is multiplied by the output of the SoftMax to find the weighted input. This operation is expressed in the following formula:

(1) $$Attention\left(Q,K,V\right)=Softmax\left(\frac{QK^{T}}{\sqrt{d_{K}}}\right)\cdot V,$$

Multiple independent self-attention heads compute the scaled dot product attention in the MSA block. The results of all the attention heads are concatenated together and then passed to the FFN block. The FFN consists of two fully connected layers with a Gaussian error linear unit activation function (GELU) applied in between [58]. The encoded image representation obtained from the image encoder is subsequently projected into a vector of dimension 512 to match it with the dimension of the question representation. Thus, the resultant representation $f_{Xi}$ has a dimension of 49 × 512. 

### 3.2. Question Encoder 

**Figure 3:**
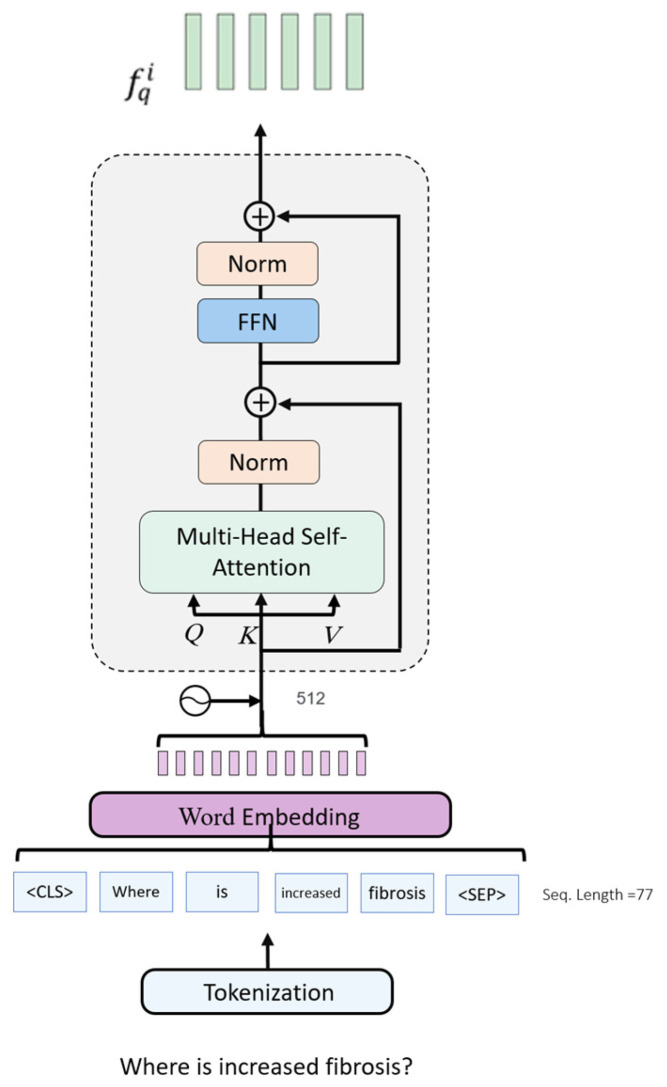
The architecture of the text encoder.

The question encoder uses a BERT-like architecture to generate the question’s textual features [59]. Similar to the image encoder, the question encoder consists of a stack of 12 identical layers. As shown in Figure 3, the first step in encoding the question is tokenization, in which the question is tokenized as a sequence of word tokens. Two special tokens, <CLS> and <SEP>, are appended to the sequence to mark its beginning and end, respectively. The encoder uses a sequence with a fixed length equal to 77 tokens and uses a vocabulary size of 49,408 words. The word embedding layer embeds the sequence of the question tokens into features of dimension 512. A learnable positional embedding is added to the sequence to provide information about the order of each word. The final representation is generated by feeding the initial representation through the 12 layers of the encoder. Analogously to the image encoder, the question encoder employs the MSA block to capture dependencies within the question tokens. The model also uses normalization layers and skip connections, but unlike the image encoder, the normalization layers come after the MSA and FNN. The output of the question encoder is the question feature representation of size 77 × 512. This representation holds information about the semantics of the question and the relationships between words.

### 3.3. Multi-Modal Representations

Our VQA model is supposed to receive a question and look at the given image to find relevant information for generating the correct answer. To model this, the image features $f_{Xi}\in R^{49\times 512}$ obtained from the image encoder and the question features $f_{qi}\in R^{77\times 512}$ obtained from the question encoder are concatenated to form the joint representation $f^{i}=f_{X}^{i}⊕f_{q}^{i}$. Here, ⊕ is the concatenation operator. The representation $f^{i}$, which aggregates the relevant information from the two modalities, is supplied as input to the answer generator which decodes it into an answer. 

Since the VQA task requires encoding both the question and the image, we leverage the rich semantic embedding of the contrastive language–image pre-training (CLIP) model [52] and use it as a backbone. CLIP is built on dual transformers that have been optimized by contrastive learning to match a large batch of image–text pairs. Specifically, CLIP learns a multi-modal embedding space by jointly training an image encoder and a text encoder on a corpus of 400 M image–text pairs. The contrastive learning used by the CLIP model aims at maximizing the similarity of truly corresponding image–text pairs while minimizing the similarity of mismatched image–text pairs.

### 3.4. Answer Decoder 

**Figure 4:**
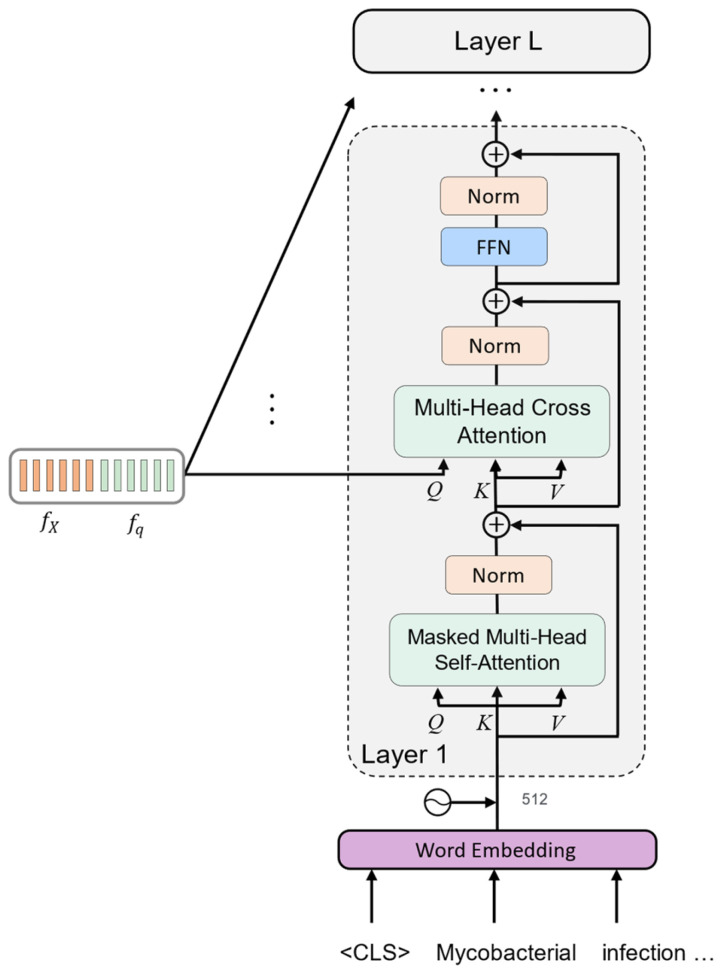
The architecture of the answer decoder.

The decoder is modeled as a generative model. It generates the answer one word at a time in an autoregressive manner. When a word is predicted, it is added to the input sequence, which then serves as the model’s new input in the next time step. The decoder architecture consists of two identical layers. Figure 4 shows the internal architecture of a single layer. Similar to the question encoding, the input answer is first tokenized into words and trimmed or padded to the maximum length of 77 words. The two special tokens <CLS> and <SEP> are appended to the sequence, and each word is represented as a word embedding. The positional information is added, and the word is fed into the first layer of the decoder. The decoder layer is composed of the same MSA and FFN blocks present in the encoder. However, the decoder uses a masked self-attention block that learns the dependencies within the answer tokens without considering future tokens. This helps the model to make a prediction about the next word based on the sequence of the previous tokens. Another difference in the decoder is the multi-head cross-attention block, which is designed to capture the interdependencies between two different inputs, as opposed to the self-attention mechanism employed by the image and question encoders, which derives *Q*, *K*, and *V* from the same modality. As shown in Figure 4, the cross-attention mechanism in each decoder layer uses *Q* derived from the multi-modal representation, and *K*, and *V* derived from the answer. This helps the model to detect the correlation between the different data modalities involved in the VQA task.

### 3.5. Network Optimization

Let us consider $f_{Xi}$ and $f_{qi}$ as the visual and textual representations of the image $X_{i}$ and the question $q_{i}$, respectively, with $f_{Xi}\in R^{49\times 512}$ and $f_{qi}\in R^{77\times 512}$. The 49 and 77 represent the length of the image and question sequences, respectively, and 512 represents the feature dimension. The two features are generated by fixing the weights of the CLIP encoders. The goal of training is to fine-tune the parameters of the answer decoder by optimizing it on the autoregressive language modeling objective function conditioned on the multi-modal joint feature representation $f_{i}$ and the previously generated tokens of the answer. Formally, the objective function is defined as:

(2) $$L=-\sum_{k=1}^{K}{logP(a}_{i,k}|a_{i,0}\ldots a_{i,k-1},f_{i}),$$

where $f_{i}$ is the joint feature representation of the image $X_{i}$ and the question $q_{i}$, ${a_{i,0},\ldots ,a}_{i,k-1},a_{i,k}$ are the words composing the answer $y_{i}$, and K is the number of words in the answer $y_{i}$.

At the inference phase, the test image and the question are given as input to the image encoder and the question encoder, respectively, to obtain the joint feature representation. The decoding begins by reading this representation and the start token to generate the first word of the answer. The model generates a word at each step by sampling the word with the highest posterior probability over the vocabulary V. The predicted words constitute the answer to the question about the test image. The answer generation is terminated when either the end of the sequence token is predicted, or the maximum sequence length is reached.

## 4. Experimental Results

In this section, we first introduce the datasets and then explain the evaluation metrics utilized in this work and the experimental setup. Lastly, we present results and analysis related to our experiments.

### 4.1. Dataset Description

**Figure 5:**
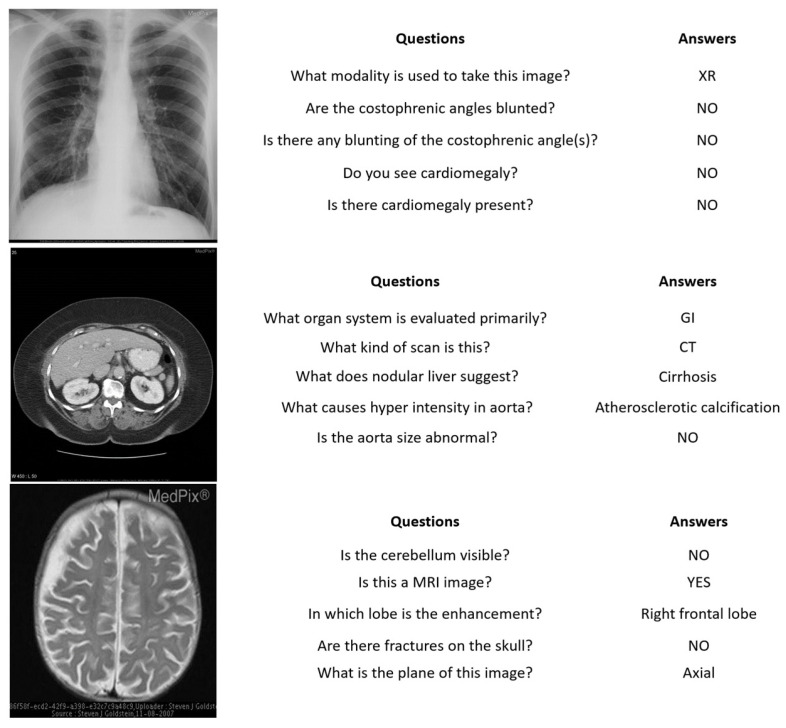
VQA-RAD images, questions, and answers.

**Figure 6:**
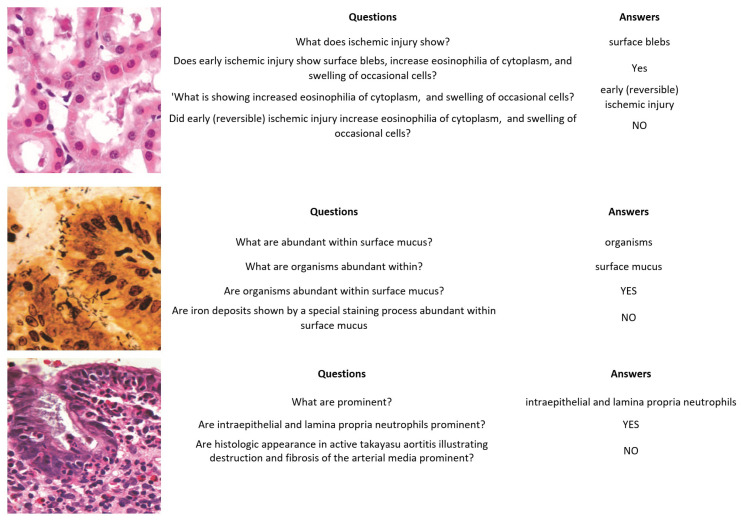
PathVQA images, questions, and answers.

We trained and evaluated our model on two medical VQA datasets, namely VQA-RAD and PathVQA. The characteristics of these datasets are listed in Table 1, and sample images and their corresponding question–answer pairs from the training set are shown in Figure 5 and Figure 6.

- VQA-RAD [41] is a manually constructed dataset in the field of medical VQA. It contains 3515 question–answer pairs generated by clinicians and 315 radiology images that are evenly distributed over the head, chest, and abdomen. Each image is associated with multiple questions. The questions are divided into a training set and a test set which contain 3064 and 451 question–answer pairs, respectively. Questions are categorized into 11 categories: abnormality, attribute, modality, organ system, color, counting, object/condition presence, size, plane, positional reasoning, and other. Half of the answers are closed-ended (i.e., yes/no type), while the rest are open-ended with either one-word or short phrase answers.
- PathVQA [40] is the first dataset of pathology images. It contains a total of 4998 pathology images with 32,799 question–answer pairs. The dataset is split into three sets: training, validation, and test sets. The validation set has 987 images and 6279 question–answer pairs, the test sets contain 990 images with 6761 question–answer pairs, and the training set includes 3021 images with 19,755 question–answer pairs. Every image has several questions that relate to multiple aspects such as location, shape, color, appearance, etc. The questions are categorized into two types, with several varieties: open-ended questions such as why, what, how, where, etc., and closed-ended questions.

### 4.2. Evaluation Measures 

To quantitatively validate the proposed methodology and compare our results to other state-of-the-art methods, we used the metrics proposed in [60] which are a commonly used evaluation metric in VQA. We adopted strict accuracy to measure the ratio between correctly predicted observations and total observations. BiLingual Evaluation Understudy [61] (BLEU) is another automatic evaluation metric to measure the similarity of predicted answers and ground-truth by matching n-grams, as expressed below:

(3) $$BLEU=BP\cdot exp(\sum_{n=1}^{N}w_{n}\log_{e}P_{n}),$$

where BP is the brevity penalty to penalize short answers, $w_{n}$ is the weight between 0 and 1 for $\log_{e}P_{n}$ and $\sum_{n=1}^{N}w_{n}=1$, $P_{n}$ is the geometric average of the modified n-gram precision, and N is the maximum length of n-grams. N-grams here are up to length 4.

### 4.3. Experimental Setup

For the experiment, we trained our model with PyTorch [62], and experiments were run on a machine with an Intel Core (TM) i9-7920× CPU @ 2.9 GHz, RAM of 32 GB, and an NVIDIA GeForce GTX 1080 Ti Graphical Processing Unit (GPU) (with 11 GB GDDR5X memory). During our experiments, we defined the following hyperparameters. The input and output dimensions of the multi-head attention are 512, the dropout in all full connection layers is 0.1, and we set two transformer blocks in the encoder and decoder. As for training, we used the Adam optimizer [63] with a base learning rate set to 0.001. The batch size for training was set to 50, and the number of training epochs was set to 50. The images were shuffled for each epoch, and randomly flipped left and right with a probability of 0.2. We used the BERT-base-uncased tokenizer for text inputs with vocabulary size 49,408.

### 4.4. Results

The evaluation results of the visual question answering (VQA) model using the VQA-RAD and the PathVQA datasets are presented in Table 2. Several metrics are used to assess the model’s performance, including BLEU scores and both open-ended and closed-ended accuracy ratings by using two decoder layers. For the VQA-RAD dataset, the BLEU-1 test resulted in the highest score of 71.03%, while the BLEU-4 test resulted in the lowest score of 64.43%. The score for closed-ended accuracy is 82.47%, and the score for open-ended accuracy is 71.49%. The model’s overall accuracy is 75.41%. The results on the PathVQA dataset are also shown in Table 2, and the model’s effectiveness is assessed with different measures. The BLEU-1 test resulted in the highest score of 61.78%, while the BLEU-4 test resulted in the lowest score of 58.19%. The score for closed-ended accuracy is 84.63% while the score for open-ended accuracy is 58.29%. The model’s total accuracy is 67.05%.

Overall, the results show that the model performs well on both datasets. The closed-ended accuracy metric received the greatest results, demonstrating the model’s superior ability to choose the right response from a pre-defined set of options. The model may have trouble producing free-form replies, as indicated by the lower results on the open-ended accuracy metric. The lower BLEU scores imply that there may be less overlap between the model’s predictions and the ground-truth answers.

### 4.5. Discussions

Table 3 shows the effect of using a different number of layers in the answer decoder on the two datasets, RAD-VQA and PathVQA. The results of the VQA-RAD dataset exhibit a consistent improvement in all measures as the number of layers increases. For the PathVQA dataset, the highest results are obtained with three decoder layers, while in the case of the VQA-RAD dataset the best results are obtained using four decoder layers. For quantifying the capability of the proposed model, we contrast our results against several models. For instance, Table 4 compares the results of open-ended questions with the main work that introduces the PathVQA dataset. We recall that these metrics are not used in most recent works. The results are shown in terms of BLEU scores (1, 2, and 3), as well as the F1 score. It is clear that our approach yields superior outcomes thanks to its autoregressive decoder that is able to generate answers in the form of sentences. 

In Table 5, we also compare our results to several existing state-of-the-art approaches [6,7,43,45,46,64,65] in terms of open-ended and closed-ended accuracies. For instance, in [6] the authors suggest a framework that uses conditional reasoning to automatically learn how to reason effectively for different VQA-RAD tasks. Their approach involves creating a reasoning module that considers the question being asked and uses this information to select the most important features from a fusion of different types of data. The authors of [7] suggest a system that tackles the issue of limited labeled data by means of an unsupervised denoising autoencoder and supervised meta-learning. The denoising autoencoder helps to make use of a large number of unlabeled images, while meta-learning helps learn meta-weights that can quickly adapt to VQA problems that have limited labeled data. On the other side, the authors of [45] propose a cross-modal self-attention (CMSA) module that enhances the fusion of visual and linguistic features by capturing long-range contextual relevance. This module helps to better incorporate information from both visual and linguistic modalities by emphasizing their important and relevant features. In another work [43], the authors introduce a method for quantifying multiple meta-models that utilizes meaningful features and learns meta-annotations. Finally, in [64], the authors suggest a data augmentation method called VQAMix to address the data limitation problem in VQA.

Compared to all these models, our method shows improvements for the metrics closed, open, and overall accuracy. Specifically, we observed an improvement of about 2.5%, 11%, and 5% in VQA-RAD and 2%, 58%, and 18% in PathVQA. We also noticed that our model’s improvements were more significant on open-ended questions compared to yes/no questions.

**Figure 7:**
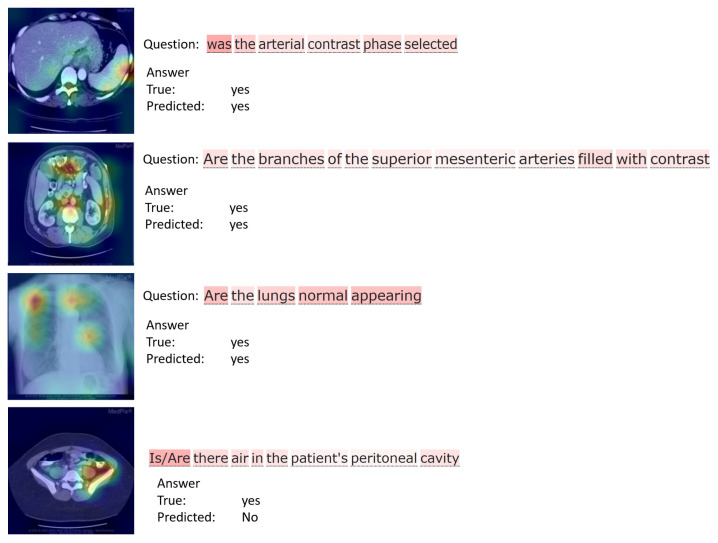
Attention maps obtained for sample images from RAD-VQA dataset.

**Figure 8:**
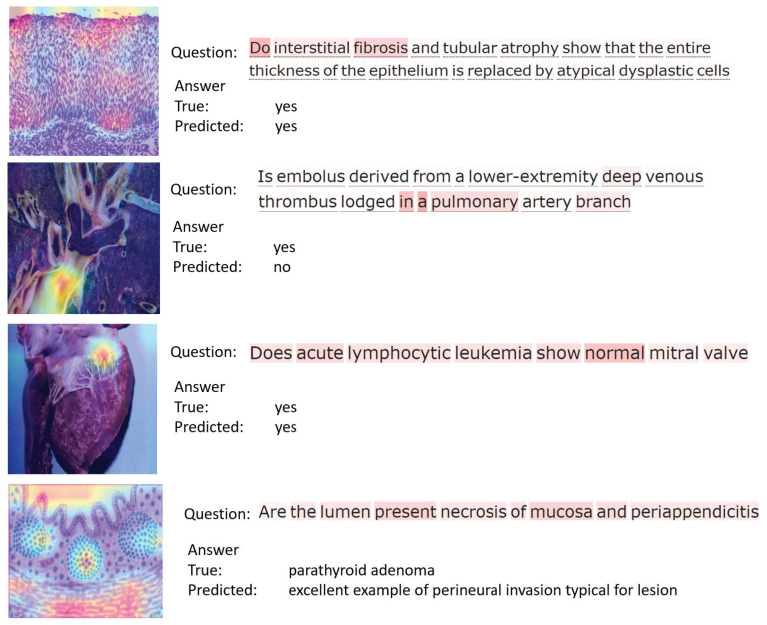
Attention maps obtained for sample images from PathVQA dataset.

For further analysis, we present in Figure 7 and Figure 8 the attention maps of questions and images obtained for samples from both datasets. These maps are useful to understand the regions of interest of an image and the corresponding question. They can also help radiologists and other medical professionals to identify key regions of an image, and to improve the performance of automated diagnostic systems.

Considering the RAD-VQA dataset samples displayed in Figure 7, for the first two samples the model provides the correct answers, and the attention maps highlight the “arterial” region in the first image and the “mesenteric arteries” in the second image. The third example also shows a correctly predicted answer, with the corresponding attention map placing emphasis on the lung area. This is consistent with the question that concerns a general query about lung condition. The last sample shows a failure case in which the model could not predict the correct answer about the “peritoneal cavity”.

Figure 8 shows four samples of questions answered by our model for images from the PathVQA dataset. The first sample shows that the model correctly predicts the answer and the attention span across the relevant regions in the image. In the second example, although the model cannot provide the correct answer, it can still highlight related regions in the image. The third sample asks about the condition of the “mitral valve”. The question is correctly answered by our model and the corresponding region in the image is highlighted. Finally, the question asked in the fourth example is an open-ended question, regarding the “lumen” present in the image. It can be seen that the model could not obtain the correct answer because open-ended questions are more challenging, and require further developments.

## 5. Conclusions and Future Work

In this work, we have proposed a VQA model for medical datasets. This model is composed of an image and text encoders for encoding the medical image and the corresponding question. Then, a decoder is mounted on the top to generate an answer in an autoregressive way. The complete architecture is end-to-end learnable via the backpropagation algorithm. In the experiments, we have validated our model on two well-known VQA medical datasets, namely VQA-RAD and PathVQA. The obtained results confirm its promising capabilities compared to recent works. For future developments, we will look for more effective data augmentation methods to achieve better performance. In addition, we will investigate other types of multi-modal transformers to learn better representations for both images and textual questions.

## Figures and Tables

**Table 1:** Medical VQA datasets.

Dataset	No. of Images	No. of Question/Answer Pairs	Question Types
VQA-RAD	315	3515	open-ended, closed-ended
PathVQA	4998	32,799	

**Table 2:** BLEU-1, 2, 3, 4, closed-ended, open-ended, and overall accuracies.

Dataset	Evaluation Metric						
	B1	B2	B3	B4	Closed	Open	All
VQA-RAD	71.03 ± 0.90	70.81 ± 0.95	67.01 ± 0.99	64.43 ± 0.99	82.47 ± 1.43	71.49 ± 0.73	75.41 ± 0.98
PathVQA	61.78 ± 0.03	61.16 ± 0.04	59.28 ± 0.02	58.19 ± 0.02	84.63 ± 0.83	58.29 ± 0.73	67.05 ± 0.58

**Table 3:** Sensitivity analysis with respect to the number of decoder layers.

#Decoder Layers	VQA-RAD Dataset			PathVQA Dataset		
	Closed	Open	All	Closed	Open	All
1	82.06 ± 1.21	70.98 ± 0.39	74.99 ± 0.85	83.13 ± 0.49	57.90 ± 0.49	66.17 ± 0.75
2	82.47 ± 1.43	71.49 ± 0.73	75.41 ± 0.98	84.63 ± 0.83	58.29 ± 0.73	67.05 ± 0.58
3	84.46 ± 1.43	72.51 ± 0.99	76.78 ± 1.09	86.90 ± 0.21	62.58 ± 0.04	70.65 ± 0.07
4	84.99 ± 1.15	72.97 ± 1.49	77.27 ± 1.37	86.83 ± 0.31	62.37 ± 0.22	70.54 ± 0.25

**Table 4:** BLEU-1, 2, 3 and F1 on open-ended questions.

Method	Evaluation Metric			
	BLEU-1	BLEU-2	BLEU-3	F1 (%)
GRU + Faster R-CNN [40]	32.4	22.8	17.4	24.0
CNN + LSTM [40]	13.3	9.5	6.8	12.5
SAN+ CNN + LSTM [40]	19.2	17.9	15.8	19.7
SAN+ CNN + LSTM+ Faster R-CNN [40]	24.7	19.1	16.5	21.2
SAN+ CNN + LSTM+ ResNet [40]	19.9	18.0	16.0	19.8
Proposed	71.03 ± 0.90	70.81 ± 0.95	67.01 ± 0.99	72.85 ± 0.95

**Table 5:** Comparison with state-of-the-art methods.

Method	VQA-RAD Dataset			PathVQA Dataset		
	Closed	Open	All	Closed	Open	All
Zhan, L.M. et al. [6]	79.3	60.0	68.5	-	-	-
Nguyen, B.D. et al. [7]	75.1	43.9	62.6	81.4	8.1	44.8
Gong, H. et al. [45]	77.8	52.8	67.9	-	-	-
Do, T. et al. [43]	72.4	52.0	64.3	82.1 ± 0.5	11.8 ± 0.6	47.1 ± 0.4
Gong, H. et al. [64]	69.7	38.2	57.1	75.3	5.4	40.5
Gong, H. et al. [64]	72.4	49.6	63.3	81.3 ± 0.3	9.1 ± 0.5	45.3 ± 0.4
Gong, H. et al. [64]	79.6	56.6	70.4	83.5 ± 0.2	13.4 ± 0.6	48.6 ± 0.3
Moon, J.H. [65]	77.7 ± 0.71	59.5 ± 0.32	-	-	-	-
Proposed	82.47 ± 1.43	71.49 ± 0.73	75.41 ± 0.98	85.61 ± 0.83	71.49 ± 0.73	66.68 ± 0.58

## Data Availability

The data presented in this study are available on request.
